# Automated Analysis of 1p/19q Status by FISH in Oligodendroglial Tumors: Rationale and Proposal of an Algorithm

**DOI:** 10.1371/journal.pone.0132125

**Published:** 2015-07-02

**Authors:** Céline Duval, Marie de Tayrac, Karine Michaud, Florian Cabillic, Claudie Paquet, Peter Vincent Gould, Stéphan Saikali

**Affiliations:** 1 Department of pathology, Centre Hospitalier Universitaire de Québec, Québec, Canada; 2 Department of genomic and molecular genetics, Centre Hospitalier Universitaire de Rennes, Rennes, France; 3 Department of Neurosurgery, Centre Hospitalier Universitaire de Québec, Québec, Canada; 4 Department of cytogenetics and cellular biology, Centre Hospitalier Universitaire de Rennes, Rennes, France; University of Torino, ITALY

## Abstract

**Objective:**

To propose a new algorithm facilitating automated analysis of 1p and 19q status by FISH technique in oligodendroglial tumors with software packages available in the majority of institutions using this technique.

**Methods:**

We documented all green/red (G/R) probe signal combinations in a retrospective series of 53 oligodendroglial tumors according to literature guidelines (Algorithm 1) and selected only the most significant combinations for a new algorithm (Algorithm 2). This second algorithm was then validated on a prospective internal series of 45 oligodendroglial tumors and on an external series of 36 gliomas.

**Results:**

Algorithm 2 utilizes 24 G/R combinations which represent less than 40% of combinations observed with Algorithm 1. The new algorithm excludes some common G/R combinations (1/1, 3/2) and redefines the place of others (defining 1/2 as compatible with normal and 3/3, 4/4 and 5/5 as compatible with imbalanced chromosomal status). The new algorithm uses the combination + ratio method of signal probe analysis to give the best concordance between manual and automated analysis on samples of 100 tumor cells (91% concordance for 1p and 89% concordance for 19q) and full concordance on samples of 200 tumor cells. This highlights the value of automated analysis as a means to identify cases in which a larger number of tumor cells should be studied by manual analysis. Validation of this algorithm on a second series from another institution showed a satisfactory concordance (89%, κ = 0.8).

**Conclusion:**

Our algorithm can be easily implemented on all existing FISH analysis software platforms and should facilitate multicentric evaluation and standardization of 1p/19q assessment in gliomas with reduction of the professional and technical time required.

## Introduction

Determination of chromosome 1p and 19q status has become an important step in the diagnosis [1,2] and the management of oligodendroglial tumors [3–9]. Codeletion of 1p and 19q whole arms appears strongly correlated with a better response to standard treatment with radiotherapy and chemotherapy as well as a better overall survival [10–14]. Several molecular techniques are available to study 1p/19q status among which the fluorescence *in situ* hybridization (FISH) technique is one of the most widespread giving its easy implementation on paraffin embedded tumor tissue [15–19]. One of the major advantages of FISH lies in its ability to preselect the analyzed tissue morphology and to allow the study of large tissue areas when necessary. Nevertheless, manual FISH analysis is time-consuming and depends on subjective interpretation by the reader. These difficulties are overcome by the automated evaluation of signal patterns using FISH analysis software. The first reports of automated FISH analysis were published in the early 90s on isolated cells [20,21]. Since then, many advances, including automated evaluation of histological specimens, have been achieved, and in the last decade many clinical applications of automated FISH analysis in the field of cancer cytogenetics have been published, including in breast [22,23] and lung tumors [24] and in hematological malignancies [25,26].

Despite the fact that all commercial FISH platforms are equipped with automated analysis software, only one article exists in the literature concerning the automated analysis of 1p and 19q status in oligodendroglial tumors [27] and there are still no specific guidelines as to the automated interpretation of FISH results, unlike those which exist for manual analysis, although the latter are themselves imprecise [28,29]. In automated analysis several parameters can be set in the image analysis software including the size of the cell or the nucleus and the size or intensity of the chromosomal fluorescent probe signal, although these parameters are usually determined by the manufacturer and do not require a specific pre-setting. The major parameter most often used in manual analysis, which therefore has to be well defined in complementary and comparative automated analysis, is the definition of which green “G” (reference) / red “R” (marker) signal combinations should be taken into account for the FISH analysis. In the absence of specific guidelines, FISH automated analysis for 1p/19q status typically uses the same criteria as for the manual analysis as defined in the guidelines of the International Society of Paediatric Oncology (ISPO) for studies of peripheral neuroblastic tumours [28]. The latter defines an open analysis grid allowing the classification of any combination encountered into 3 categories of chromosomal status (deletion, normal or imbalanced status) but it was originally proposed for analysis of intact nuclei on touch preparations, so its application to paraffin-embedded tissue sections with inevitable nuclear truncation artefacts remains poorly documented and of uncertain validity.

The aim of our study was to define an algorithm allowing for satisfactory concordance between manual and automated analysis of 1p/19q status by FISH and to improve this algorithm by using the 3 different ways most often used in the literature to calculate the final 1p/19q status (the combination method, the ratio method and the combination + ratio method) in order to permit the widest possible comparison with the literature data, and to determine which of these methods allows for the best concordance between manual and automated analysis.

**Fig 1:**
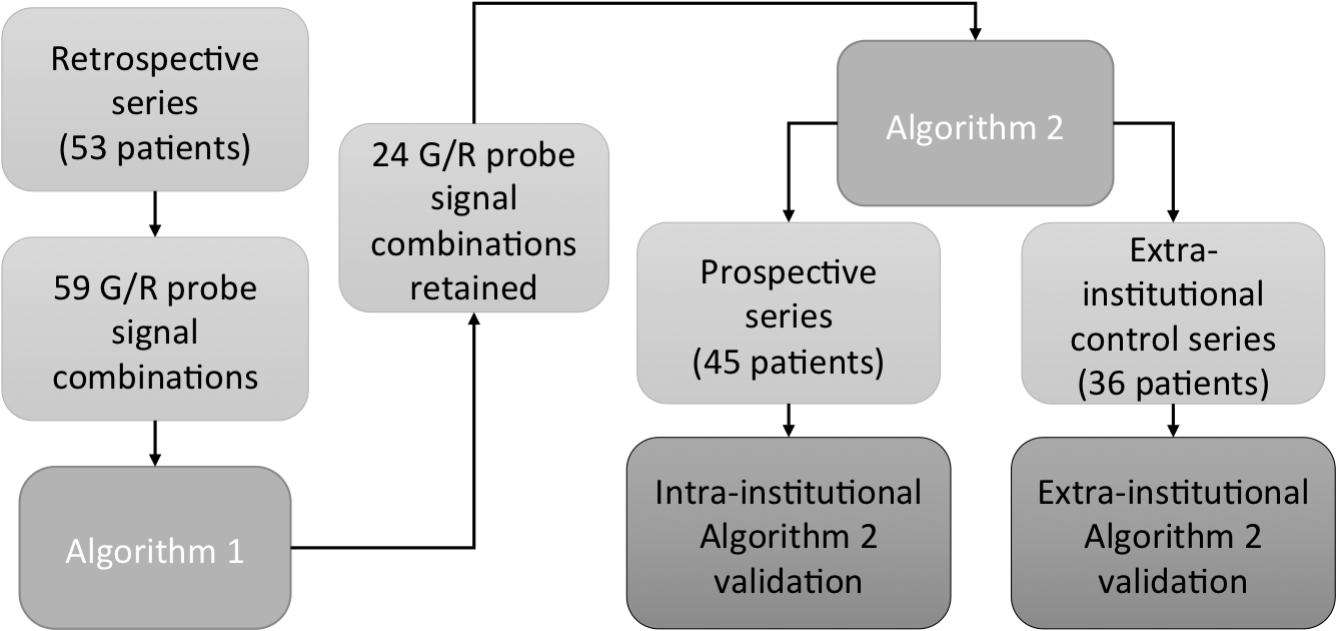
Flow-chart detailing the different steps in the construction and validation of Algorithm 2. Algorithm 2 only includes G/R combinations which appeared statistically correlated to one of the three chromosomal status subgroups (deletion, normal or imbalance for 1p and/or 19q) in the retrospective series.

In a first step, manual and automated analysis of 1p/19q status was performed on a retrospective series of 53 oligodendroglial tumors using the published ISPO criteria and listing all observed combinations, which allowed us to define Algorithm 1. In a second step, only those G/R combinations defined in Algorithm 1 which appeared statistically correlated to one of the three chromosomal status subgroups defined in our study (deleted, normal or imbalanced for 1p and/or 19q) were retained in a new more stringent algorithm (Algorithm 2). Algorithm 2 was then tested and refined on a prospective internal series of 45 oligodendroglial tumors, paying particular attention to the concordance between manual and automated analysis. Finally Algorithm 2 was tested on an external series of 36 gliomas in order to determine the level of concordance between the initial manual analysis and the second control automated analysis (Fig 1).

## Materials and Methods

### Ethics statement

Our IRB (Research Ethics Committee of the Centre Hospitalier Universitaire de Québec) was consulted regarding this study and waived study approval and consent, the aim of this study being the optimization of an institutional diagnostic technique with anonymized data (notice 2016–2455): S1 Table. Tumor samples were collected and anonymized by the Pathology Service of the Centre Hospitalier Universitaire de Québec (Hôpital de l’Enfant-Jésus, Quebec City, Canada).

### Patients and tissue specimens

Formalin fixed paraffin-embedded (FFPE) tissue from two series of 53 and 45 consecutive oligodendroglial brain tumor samples (biopsies or surgical resections) studied in our institution between 2006 and 2011 and between 2011 and 2014 respectively was selected for the retrospective and prospective FISH studies. All tumors were classified and graded according to the guidelines of the World Health Organization [30] by two neuropathologists (PVG and SS). For the retrospective series, cases included 11 WHO grade II oligodendrogliomas (OII), 19 WHO grade III anaplastic oligodendrogliomas (OIII), 13 grade II oligoastrocytoma (OAII), 7 grade III oligoastrocytoma (OAIII) and 3 WHO grade IV glioblastomas with oligodendroglial component (GBMO). For the prospective series, cases included 7 OII, 11 OIII, 15 OAII, 11 OAIII and 1 GBMO (Table 1). An external control series of 36 gliomas and glioneuronal tumors surgically removed between 2010 and 2015 at the Centre Hospitalier Universitaire de Rennes (France) was provided by the cellular biology service of that hospital. These cases included 1 pilocytic astrocytoma (PA), 1 Dysembryoplastic Neuroepithelial Tumour (DNT), 6 OII, 1 OIII, 5 OAII, 13 OAIII, 2 OA IV, 6 GBMO and 1 anaplastic astrocytoma (AIII): S2 Table.

**Table 1:** Clinical, histological and chromosome status data for the retrospective and prospective series.

	Number	Mean Age	Sex		Histology					Site		
Retrospective	(%)	(range)	M	F	OII	OIII	OAII	OAIII	GBMO	F	T	Other
Codeletion	20 (37)	45 (26–63)	12	8	7	9	2	2	0	16	3	1
Deletion 1p	3 (6)	76 (74–79)	2	1	0	3	0	0	0	1	1	1
Deletion 19q	3 (6)	59 (54–62)	1	2	0	1	0	1	1	1	2	0
Normal	8 (15)	50 (25–82)	3	5	0	3	3	1	1	5	2	1
Imbalance	19 (36)	48 (23–76)	11	8	4	3	8	3	1	7	7	5
**Total (%)**	**53**	**50**	**29 (55)**	**24 (45)**	**11 (20)**	**19 (36)**	**13 (25)**	**7 (13)**	**3 (6)**	**30 (57)**	**15 (28)**	**8 (15)**
**Prospective**			** **	** **	** **	** **	** **	** **	** **	** **	** **	** **
Codeletion	19 (42)	52 (26–77)	7	12	5	8	2	3	1	14	3	2
Deletion 1p	2 (4)	51 (42–60)	1	1	1	0	0	1	0	1	1	0
Deletion 19q	2 (4)	40 (28–52)	2	0	1	0	1	0	0	0	1	1
Normal	5 (12)	52 (25–86)	2	3	0	0	4	1	0	1	2	2
Imbalance	17 (38)	44 (24–63)	6	11	0	3	8	6	0	11	2	4
**Total (%)**	**45**	**48**	**18 (40)**	**27 (60)**	**7 (16)**	**11 (24)**	**15 (34)**	**11 (24)**	**1 (2)**	**27 (60)**	**9 (20)**	**9 (20)**

OII: WHO grade II oligodendroglioma, OIII: anaplastic oligodendroglioma

OAII: grade II oligoastrocytoma, OAIII: anaplastic oligoastrocytoma

GBMO: glioblastoma with oligodendroglial component, F: frontal, T: temporal.

### FISH technique

FISH analysis of 1p/19q status was performed using the LSI 1p36/1q25 and 19q13/19p13 Dual-Color Probe kit (Abbott Molecular Inc., Abbott Park, Illinois, USA). Briefly, 5-μm-thick formalin-fixed, paraffin-embedded sections were deparaffinized, treated with saline sodium citrate and digested in pepsin solution. The probe mix (5 to 15 μl) was added to each slide according to the manufacturer’s instructions. Target DNA and probes were codenatured at 74°C for 5 minutes and incubated at 37°C overnight in a humidified hybridization chamber (ThermoBrite, Abbott Molecular Inc.). Post-hybridization washes were performed in NP40 0.3%/2×SSC (pH 7) at 75°C for 2 minutes. Finally, the slides were air dried and counterstained with DAPI (4′,6-diamidino-2-phenylindole) diluted in Vectashield (Vector, Burlingame, CA, USA). Signal acquisition was performed for each slide over 8 and 12 more representative areas for retrospective and prospective series respectively. These areas were automatically captured at x400 using a Metasystem station (Zeiss MetaSystems, Thornwood, NY) equipped with a Zeiss Axioplan fluorescent microscope. These acquired images were then used as the basis for the manual and the automated counting assays. Automated analysis was performed using the Metafer 4 software (Metasystem). Cases from the external control series were previously treated by a similar procedure using the same probes and our automated analysis was performed on archival slides which had been stored at -20°C.

### FISH interpretation

FISH manual analysis was performed by two independent observers (CD for the retrospective series and SS for the prospective series). For each case, observers assessed 100 non-overlapping nuclei for green ‘G’ (reference) and red ‘R’ (marker) signals. Automatic analysis was performed on all tumor cells identified by the Metafer 4 software using the "1p19q tile-sampling classifier" provided by this software. This sampled a mean of 333 cells (min: 115 – max: 643 – median: 315) for the retrospective series and 574 cells (min: 119 – max: 1506 – median: 600) for the prospective series.

Three methods of counting the signals were used: the combination method, the ratio method and the combination + ratio method. For the combination method, the frequencies of signal patterns for 1q (G) and 1p (R) on one slide, and for 19p (G) and 19q (R) on another slide, were noted.

To facilitate comparison with reference guidelines in the literature [27–29], we indentified our combinations as the ratio of the green probe to the red probe (G/R) and not vice versa as done with the ratio method.

For Algorithm 1, G/R combinations were briefly classified as follow [16,28]:

- Simple deletion: ≤ one signal for R and R/G < 0.5 (2/1, 3/1, 4/1, etc….)
- Relative deletion: deletion in a background of polysomy and R/G < 0.5 (4/2, 6/3, etc…)
- Normal: 1p/19q intact and R/G = 1 (2/2)
- Imbalanced: everything else and R/G > 0.5 (4/3, 4/4, 4/5, etc…)

For Algorithm 2, G/R combinations were defined as detailed in Results.

The cut-off value for the number of nuclei that had to show deletion in order to be considered abnormal was calculated on a series of 13 non-neoplastic brain tissue samples (from epilepsy surgery cases and normal autopsy brains). This cut-off was calculated using mean +3 SD and was set at 55% for both 1p and 19q for deletion status and 30% for imbalanced status using the Algorithm 1 and at 55% and 20% respectively using the Algorithm 2. A tumour was therefore classified as deleted if the percentage (%) of deleted nuclei exceeded 55%. In the other cases a tumor was classified as (1) normal if the percentage of deleted plus imbalanced nuclei was less than the cut-off value or (2) imbalanced if the sum of imbalanced + deleted nuclei was greater than or equal to the cut-off value [27,28]. Cases with over 30% of imbalanced nuclei (which correspond to mean +3 SD in normal tissue control) were considered imbalanced independently of their deletion status. For automated analysis, interpretation was the same as for manual analysis and with the same deletion and imbalance cut-offs as defined for Algorithm 1 and 2 respectively. For the ratio method, the signal ratio of red signals to green signals was calculated for each case. A ratio ≤ 0.8 was considered to indicate a deletion whereas a ratio between 0.8 and 1.15 was considered to indicate a normal status on the chromosomal arm. A ratio over 1.15 was considered to indicate polysomy and was classified in the imbalanced status subgroup [4,31]. The combination + ratio method combines the results of the two previous methods. Cases deleted in one method and normal in the other were considered as deleted. Cases imbalanced in one method and deleted or normal in the other were considered as imbalanced.

### Statistical analyses

All statistical analyses were carried out with the R statistical environment (http://www.R-project.org/). Association between the different G/R combinations identified during manual and automated analysis and the chromosomal status was studied on retrospective and prospective series using logistic regression models. Concordance between manual analysis and automated analysis was estimated by calculating Cohen's kappa coefficient (κ) with the Kappa function of the R package *vcd*. We considered that a κ value between 0.6 and 0.8 reflected good agreement and that a value >0.8 constitutes high concordance. A Chi-square test was performed for group comparisons between clinical, histological and chromosomal status data for the retrospective and prospective populations. This test was also used for group comparisons between 1p and 19q chromosomal status obtained by each of the manual and automated analysis, using the three methods of counting. p values < 0.05 were considered as significant.

## Results

### Clinical data

Age, sex, histological grading, tumor location and chromosomal status obtained by automated analysis using the combination + ratio method are summarized in Table 1 for both the retrospective and prospective series from our institution.

In the retrospective series, 41 patients underwent open surgery with gross total or partial tumor resection (n = 41; 77%) and 12 underwent stereotactic biopsies (n = 12; 23%). For purposes of statistical analysis the tumor location was considered to be the lobe of the brain within which the largest volume of the glioma resided, even if several lobes were affected. 10/53 cases were recurrent tumors (19%). In the prospective series, 40 patients (88%) underwent gross total or partial tumor resection and 5 (12%) underwent stereotactic biopsies. 9/45 cases were recurrent tumors (20%). There was a significant difference in patient sex and mean age between the two series according to 1p/19q chromosomal status (p = 0.04 and 0.0001 respectively with the Chi-square test). On the other hand, there were no significant differences in chromosomal status or tumor location or histological subtypes distributions.

The frequency of 1p/19q alterations was not significantly correlated with the WHO Grade, the sex or the age in either series. 1p/19q co-deletion was correlated with pure oligodendroglial tumors: 53% of oligodendrogliomas versus 17% of oligoastrocytomas and GBMO in retrospective series and 72% versus 22% in prospective series (p = 0.006 and p = 0.03 respectively). Co-deletion was also correlated with frontal lobe location of the tumor (p = 0.005 and p = 0.03 respectively).

### Type and frequency of G/R combinations defining Algorithm 1

The analysis of 1p and 19q chromosomal status was done on the retrospective series using Algorithm 1. Many combinations were noted on both manual and automated analysis with more combinations observed by automated versus manual analysis (42 G/R combinations versus 58 for 1p and 59 versus 40 for 19q): Table 2. Regardless of the chromosomal arm or type of analysis, the large majority of these combinations (between 65% and 81% of them) were found in less than 1% of the neoplastic cells. Only a few of these combinations (between 9% to 35%) were found in more than 1% of the neoplastic cells and only six combinations were found in more than 5% of the neoplastic cells, which accounted for 10% to 15% of total combinations. These G/R combinations are the following: 1/2, 3/1, 3/2, 1/1, 2/2, 2/1, the latter combination being the most frequently encountered in our series (Table 2).

**Table 2:** Type and frequency of G/R combinations noted during manual and automated analysis on the retrospective series.

**% of neoplastic cells**		**Manual 1p counting repartition**									**Total**	** **	**Automated 1p counting repartition**										**Total**
**<1%**	0/1	0/2*	0/3*	1/4*	2/4*	3/0*	5/1	5/3*	5/4*	4/0		**<1%**	0/0*	0/1	0/2	0/3*	0/4	1/0*	1/3*	1/4*	2/4*	3/0	
	6/2*	6/3*	6/4*	7/2	7/3	7/4	8/4	3/5	4/5*	7/5			4/0	5/0	5/1	5/3*	5/4*	6/0	6/1*	6/2*	**6/3** *	**6/4** *	
	5/5*	4/6	5/6*	12/6	5/2*	2/5*	3/2*				**28**		5/7	7/1	7/2	7/3	7/4	3/6	4/6	8/2*	8/4	8/6*	
													5/5	9/1	9/3	9/4*	9/5*	1/5	2/5*	3/5*	4/5*	12/6	
													5/6*	6/5*	7/5*								**44**
**1–5%**	2/0*	2/3*	3/3*	3/4*	4/1*	4/2*	4/3*	4/4*	1/0*	1/3*	**10**	**1–5%**	2/0*	2/3*	3/3*	3/4*	4/1*	4/2*	4/3*	4/4*	5/2*		**9**
**5–10%**	1/2*	3/1*									**2**	**5–10%**	1/2*	3/1*	3/2*								**3**
**10–20%**	1/1*	2/2*									**2**	**10–20%**	1/1*	2/2*									**2**
**20–30%**	2/1*										**1**	**20–30%**	2/1*										**1**
**Total**											42(*31)	**Total**											58(*37)
		**Manual 19q counting repartition**									**Total**		**Automated 19q counting repartition**										**Total**
**<1%**	0/1	0/2*	1/5	1/3*	1/4*	7/5	2/4*	5/2*	5/3	5/4*		**<1%**	0/0	0/1	0/2*	1/0	1/3*	1/4*	2/0	2/4*	3/0*	3/4*	
	3/0*	3/5*	4/0	4/5*	5/6	5/1*	5/5*	7/1	7/2	7/3			4/0*	4/4*	5/0	5/1*	5/2*	5/3*	5/4*	6/1	6/2*	6/3*	
	6/5	6/2	6/3	6/4	8/4	3/2*					**26**		6/4	7/1	7/2	7/3	7/4*	8/1	8/2*	8/3	8/4	1/5	
													2/5	3/5	4/5*	4/7	5/5*	5/6*	6/5	6/7	6/8*	7/5	
													7/6	7/7	8/5	8/6	8/7	9/6	10/3*	10/6*			
																							**48**
**1–5%**	2/3*	3/3*	4/1*	4/2*	4/3*	3/4*	4/4*	1/0	2/0*		**9**	**1–5%**	2/3*	3/3*	4/1*	4/2*	4/3*						**5**
**5–10%**	1/2*	3/1*									**2**	**5–10%**	1/2*	3/1*	3/2*								**3**
**10–20%**	1/1*										**1**	**10–20%**	2/2*										**1**
**20–30%**	2/1*	2/2*									**2**	**20–30%**	1/1*	2/1*									**2**
**Total**											40(*25)	**Total**											59(*34)

G: green signal, R: red signal

*: combinations statistically associated with a chromosomal subgroup by manual **or** automated analysis. Combinations statistically associated with a chromosomal subgroup for **both** manual **and** automated analysis are indicated in bold (27 for 1p and 23 for 19q).

When considering the combinations that are statistically correlated to at least one or more of the 3 chromosomal status subgroups, regardless of manual or automated counting, the numbers decrease significantly to 31/42 manual and 37/58 automated combinations for 1p and to 25/40 manual and 34/59 automated combinations for 19q, which represent 73% and 64% of initial combinations respectively for 1p, and 62% and 58% respectively for 19q. Among these combinations which correlate with chromosomal status, we find all (15/15) of those which are expressed by more than 1% of the tumor cells.

27/42 (64%) and 27/58 (47%) of these combinations which correlate with chromosomal status, are significant in both manual and automated counting for 1p, and in 23/40 (57%) and 23/59 (39%) for 19q. Among these combinations we find the majority (13/15) of those which are expressed by more than 1% of the tumor cells.

In our series we observed more significant combinations for the 1p probe that for the 19q probe (27 versus 23 respectively) but 19 of the significant combinations for 1p are also significant for 19q. These doubly significant combinations are generally found in the same percentage of tumor cells for both 1p and 19q.

Pooling the 19 significant combinations for both 1p and 19q, the 8 combinations for 1p alone and the 4 combinations for 19q alone allowed us to identify 31 G/R combinations which were classified in the following way according to the literature guidelines [16,28]:

- Deletion status combinations: 2/0, 2/1, 3/0, 3/1, 4/1, 4/2, 5/1, 5/2, 6/2, 6/3.
- Normal status combinations: 2/2.
- Imbalanced status combinations: 1/2, 1/3, 1/4, 2/3, 2/4, 2/5, 3/2, 3/3, 3/4, 4/3, 4/4, 4/5, 5/3, 5/4, 5/5, 5/6, 6/4.
- Unclassified combinations: 0/2, 0/3, 1/1.

### Definition of Algorithm 2

The distribution of the 31 previously identified G/R combinations was studied according to chromosomal status subgroups in the retrospective series for both 1p and 19q (Table 3). As expected, the majority of deletion status combinations G/R (2/0, 2/1, 3/0, 3/1, 4/1) and relative deletion status combinations (4/2, 5/2, 6/2, 6/3) were most often present in the deleted subgroup with a statistically significant result. The combination 5/1 was not associated with deletion status in our series. Normal status combination 2/2 was correlated with the normal subgroup. The presumed imbalanced status combination 1/2 appeared highly correlated with the normal subgroup for both 1p and 19q in our series. Combination 3/2, which has been assigned to the imbalanced subgroup by some studies [27–29] and to deletion subgroup by others [16] appeared more often in the deletion and imbalanced subgroups in our series, in comparison to the normal subgroup, but no statistical difference was found between the deletion and imbalanced subgroups for either 1p or 19q. Combinations 3/3, 4/4 and 5/5 defined as “non deleted and non imbalanced” by some studies [27,28] or as “balanced polysomies” by other [29] were correlated with the imbalanced subgroup for both 1p and/or 19q in our series.

**Table 3:** Distribution of preselected combinations used in Algorithm 2 according to chromosomal status and respective mean percentage of expression by neoplastic cells in the retrospective series.

		Deletion		Normal		Imbalance			Deletion		Normal		Imbalance	
		Mean 1p		Mean 1p		Mean 1p			Mean 19q		Mean 19q		Mean 19q	
	**G/R**	Man.	Aut.	Man.	Aut.	Man.	Aut.	**G/R**	Man.	Aut.	Man.	.Aut.	Man.	Aut.
	**0/2**	0,3	0,3	1,9	0,4	0,7	0,2	**0/2**	0	0	0,7	0	0,7	0,1
	**0/3**	-	-	-	0,1	0,1	0	**0/3**	-	-	-	-	-	-
	**1/0**	1,9	0,8	1,4	0,8	0,6	0,3	**1/0**	1,2	0,9	1	1	0,9	1
	**1/1**	13,8	17,1	13,8	16,6	6,1	14,8	**1/1**	12,7	24,7	9,4	17,8	7	17,4
Deletion	**2/0**	**2,6**	**1,8**	0,9	0,6	0,5	0,6	**2/0**	**2**	1,1	0,9	1,1	0,9	0,8
	**2/1**	**33,2**	**31,8**	11,6	12,8	9,2	12,2	**2/1**	**38,2**	**32**	12,1	15	8,7	14,3
	**3/0**	**1**	0,1	0,1	0	0,2	0,2	**3/0**	**0,2**	0,3	-	0,2	0,1	0,4
	**3/1**	**10,3**	**8,2**	0,9	1,6	3	4,4	**3/1**	**9**	**7,9**	0,8	2,8	2,9	4,7
	**4/1**	**2,2**	**2,4**	-	0,6	0,6	1	**4/1**	**2,1**	**2,4**	0,3	0,5	0,7	1,7
	**4/2**	**6,7**	**5,7**	0,4	1,8	3,8	2,8	**4/2**	**6,8**	**4,3**	1,1	1,4	2,7	2,3
	**5/1**	0,3	0,2	0,1	0,2	-	0,1	**5/1**	0,3	0,5	-	-	-	0,7
	**5/2**	**1,3**	**2**	-	0,3	0,1	0,3	**5/2**	**1**	**1,4**	-	0,1	0,1	0,2
	**6/2**	**0,6**	**0,6**	-	0,1	0,1	0,2	**6/2**	**0,2**	**0,5**	0,1	0	-	-
	**6/3**	**0,2**	**0,3**	-	0,1	0,1	0,1	**6/3**	**0,4**	**0,5**	0,1	0,1	0,1	0,1
N	**2/2**	10	11,2	**41,8**	**33,3**	23,4	19,3	**2/2**	12,3	8,1	**44,4**	**29,8**	24	19
Imbalance	**1/2**	3,7	4,1	**14,3**	**12,9**	6,9	9,5	**1/2**	3	4	**7,9**	**13,4**	6,4	10,7
	**1/3**	0,2	0,6	0,9	0,6	**2,5**	**1,6**	**1/3**	0,1	0,2	0,3	1,5	**1,6**	1,1
	**1/4**	-	0,2	-	0,1	**0,6**	**0,3**	**1/4**	-	-	-	-	**0,1**	**0,1**
	**2/3**	0,7	0,8	2,1	2,4	**5,4**	**4,4**	**2/3**	0,3	0,4	2,7	3,2	**4,1**	3,9
	**2/4**	-	0,2	0,2	0,5	**1,3**	**1,5**	**2/4**	0	0	0,5	0,3	**1,3**	**1,1**
	**2/5**	-	-	-	0	**0,3**	**0,1**	**2/5**	-	-	-	-	-	0
	**3/2**	7,3	7,2	5,4	5,8	10,1	8,2	**3/2**	7,8	7,3	4,9	3,9	9,2	9,3
	**3/3**	0,7	0,7	1,4	3	**6,1**	**5,7**	**3/3**	0,5	0,6	5,3	3,8	**7,7**	3,4
	**3/4**	0,1	0,2	0,2	1,1	**3,9**	**3,2**	**3/4**	-	0,1	1,4	0,6	**3,9**	**1,4**
	**4/3**	0,6	0,9	0,6	1,5	**4,2**	**3,2**	**4/3**	0,3	0,7	1,8	0,9	**6,6**	**1,8**
	**4/4**	0,1	0,2	0,6	1,1	**4,6**	**2,8**	**4/4**	0,1	0,1	2,3	0,9	**7**	1,3
	**4/5**	-	-	-	0,2	**0,6**	0,1	**4/5**	-	-	0,8	0,1	0,8	**0,2**
	**5/3**	0,5	0,5	0,1	0,2	0,9	0,5	**5/3**	0,3	0,5	0,3	0,3	0,6	0,5
	**5/4**	0	0,1	0,1	0,3	**1,6**	**0,5**	**5/4**	0,1	0,1	0,2	0,3	0,3	0,2
	**5/5**	0	0	-	0,1	0,1	0,1	**5/5**	-	-	0,1	0	**0,4**	**0,2**
	**5/6**	-	-	-	0	**0,1**	**0,2**	**5/6**	-	-	-	-	**0,1**	**0,1**
	**6/4**	0,1	0,4	0,1	0,1	0,2	0,3	**6/4**	0,1	0,1	-	0,2	-	0,1

Man.: manual analysis, Aut.: automated analysis, N: Normal.

Combinations which appeared statistically associated with a single subgroup are in bold.

Ten of the twelve remaining imbalanced status combinations G/R (1/3, 1/4, 2/3, 2/4, 2/5, 3/4, 4/3, 4/5, 5/4 and 5/6) were correlated with the imbalanced subgroup for both 1p and 19q. The two remaining presumed imblanced status combinations 5/3 and 6/4 were not statistically correlated with imbalanced status. Finally the three unclassified combinations G/R (0/2, 0/3 and 1/1) did not correlate with any chromosomal status in our retrospective series.

These results led us to exclude some combinations from our initial set and to propose a final algorithm (Algorithm 2) composed of 24 combinations distributed as follows:

- Deletion status combinations: 2/0, 2/1, 3/0, 3/1, 4/1, 4/2, 5/2, 6/2, 6/3.
- Normal status combinations: 2/2, 1/2.
- Imbalanced status combinations: 1/3, 1/4, 2/3, 2/4, 2/5, 3/3, 3/4, 4/3, 4/4, 4/5, 5/4, 5/5, 5/6.

### Comparison of 1p/19q status obtained by Algorithm 1 and Algorithm 2

Chromosome 1p and 19q status was calculated with Algorithm 1 on the retrospective series and with the Algorithm 2 on the prospective series. For both series, the respective algorithm was implemented for manual and automated analysis using each of the following 3 methods: the combination method, the ratio method and the combination + ratio method (Table 4). As expected in both series, the majority of the analyzed cases appeared deleted for 1p and/or 19q. Compared to the two other techniques, the ratio technique appeared to overestimate the normal cohort at the expense of the imbalanced cohort. Indeed, in the retrospective series, the combination and combination + ratio techniques report an average of 20 imbalanced cases and 9 normal cases for 1p and or 19q compared to 8 imbalanced and 18 normal cases respectively for the ratio technique. Similar results were observed with the prospective series: 17 imbalanced and 5 normal cases versus 10 and 11 cases respectively. However when analyzing only the 1p/19q deleted cohort, the ratio technique showed superimposable results to the two other techniques regardless of the algorithm used (Table 4). The 3 methods of signal analysis used in this study gave similar results for the final chromosomal status for both manual and automated analysis and for both Algorithm 1 and 2 (Table 5). The two algorithms gave similar results between manual and automated analysis when using combination or combination + ratio technique, whereas the ratio technique gave discordant results when compared to combination or combination + ratio techniques for both manual and automated analysis (Table 5).

**Table 4:** Comparison of 1p/19q status obtained by Algorithm 1 and 2 according to the counting method.

	Algorithm 1						Algorithm 2					
	Combination		Ratio		C+R		Combination		Ratio		C+R	
	Man.	Aut.	Man.	Aut.	Man.	Aut.	Man.	Aut.	Man.	Aut.	Man.	Aut.
**Codeletion**	**19**	**16**	**19**	**18**	**20**	**20**	**18**	**19**	**20**	**17**	**19**	**19**
**Deletion 1p**	**2**	**2**	**3**	**6**	**3**	**3**	**5**	**2**	**5**	**4**	**5**	**2**
**Deletion 19q**	**3**	**3**	**4**	**2**	**3**	**3**	**2**	**1**	**1**	**1**	**1**	**2**
**Normal**	**11**	**7**	**19**	**18**	**11**	**8**	**4**	**5**	**6**	**15**	**4**	**5**
**Co-imbalance**	**7**	**15**	**0**	**0**	**7**	**10**	**10**	**11**	**3**	**2**	**10**	**10**
**Imbalance 1p**	**8**	**3**	**7**	**6**	**7**	**1**	**3**	**3**	**7**	**1**	**3**	**3**
**Imbalance 19q**	**3**	**7**	**1**	**3**	**2**	**8**	**3**	**4**	**3**	**5**	**3**	**4**

C: combination, R: ratio. Algorithm 1 was studied on the retrospective series, Algorithm 2 was studied on the prospective series.

**Table 5:** Comparison of the 2 algorithms according to the type of analysis and the counting method.

	Algorithm 1			Algorithm 2		
	**C**	**R**	**C+R**	**C**	**R**	**C+R**
**Man./Aut.**	0,24	N/A	0,17	0,92	0,15	0,93
	**C / R**	**C / C+R**	**R / C+R**	**C / R**	**C / C+R**	**R / C+R**
**Man./Man.**	0,05*	0,99	0,14	0,4	0,99	0,45
	**C / R**	**C / C+R**	**R / C+R**	**C / R**	**C / C+R**	**R / C+R**
**Aut./Aut.**	0,0004*	0,84	0,0002*	0,04*	0,99	0,05*

C: combination, R: ratio, N/A: non applicable.

*: statistically significant difference (chi-squared test). Algorithm 1 was studied on the retrospective series, Algorithm 2 was studied on the prospective series.

### Concordance between manual and automated analysis according to the counting method and to the algorithm

Concordance between manual and automated analysis was studied with Algorithm 1 on the retrospective series and with Algorithm 2 on the prospective series. This study was performed with each of the methods (combination, ratio and combination + ratio) and on both 1p and 19q (Table 6). Whatever the algorithm or the counting method used, the concordance was good between manual and automated analysis and varied between 71% and 93%. Regardless of the counting method used, the correlation between manual and automated analysis varied between 77% and 87% with Algorithm 1 and between 71% and 93% with Algorithm 2.

**Table 6:** Concordance between manual and automated analysis according to the counting method and the algorithm.

	Retrospective series (Algorithm 1)		Prospective series (Algorithm 2)	
	1p	19q	1p	19q
	Manual / Automated	Manual / Automated	Manual / Automated	Manual / Automated
Combination	46/53 (87%) - 0.80	41/53 (77%) - 0.67	42/45 (93%) - 0.85	40/45 (89%) - 0.77
Ratio	43/53 (81%) - 0.69	46/53 (87%) - 0.68	34/45 (76%) - 0.63	32/45 (71%) - 0.58
Combination + Ratio	46/53 (87%) - 0.80	44/53 (83%) - 0.74	41/45 (91%) - 0.77	40/45 (89%) - 0.74
C + R (100 cells)	46/53 (87%) - 0.80	44/53 (83%) - 0.74	41/45 (91%) - 0.77	40/45 (89%) - 0.74
C + R (200 cells)	46/53 (87%) - 0.80	45/53 (85%) - 0.76	45/45 (100%) - 1	45/45 (100%) - 1

Number of cases (%) - **Κ** coefficient. C + R: Combination + Ratio.

Unlike Algorithm 1, Algorithm 2 showed a higher concordance with the combination or combination + ratio methods than with the ratio method (between 89% and 93% versus 71% and 76% respectively).

Algorithm 2 obtained a better concordance than Algorithm 1 between manual and automated analysis using the combination method (93% versus 87% for 1p and 89% versus 77% for 19q) and the combination + ratio method (91% versus 87% for 1p and 89% versus 83% for 19q). Opposite results are observed with the ratio method where Algorithm 1 gave a better concordance (81% versus 76% for 1p and 87% versus 71% for 19q).

The analysis by the combination + ratio method was extended to 200 cells in our discordant cases, which led to a total concordance of 100% with revision of the manual result in one direction from manual to automated final result when using Algorithm 2 (Table 6). Three of the discordant cases corresponded to biopsy samples, the others to surgical tumor resections.

### Validation of automated analysis by Algorithm 2 on a series from an outside institution

Validation of Algorithm 2 was carried out on an archival series of 36 gliomas from an outside institution. The same 1p and 19q probes and a FISH technique similar to ours had been used prior to archival storage at -20°C (S1 Table). In this control series, two cases were incomplete for 19q (broken slides) and 5 cases were non interpretable for 1p because of a total lack of telomeric fluorescent signal (“R” signal). 5 cases were discordant for 1p and 4 for 19q between the initial analysis and ours which represent a concordance of 84% for 1p (26/31 cases) and 94% for 19q (32/34 cases) and a total series concordance of 89% (58/65 cases) with a κ = 0.8. All discordant cases presented an important fading of signal fluorescence for the centromeric probe (“G” signal), the telomeric probe (“R” signal) or both, which led to a lack of sufficient cells identifiable by the analysis software (mean of 40 cells versus 300 for the concordant cases) and gave a final unreliable result. In all other concordant cases, although fading of the signal fluorescence was often present, leading to a decrease in the total number of analyzed cells (mean = 300, min = 150, max = 1280) compared to a newly stained slide (mean = 600, min = 220, max = 2230), this decrease was without repercussion on the final result.

## Discussion

Our study aimed to identify the most relevant chromosomal combinations for the analysis of 1p and 19q status by FISH on a retrospective series of oligodendroglial tumors and to validate these on a prospective series. Our two series did not show major bias in clinical, histological and chromosomal status data and there were no significant differences in the distribution of the chromosomal status or tumor location; the majority of the cases were co-deleted as expected from the literature [11,15,31,32]. As also expected, the majority of co-deleted cases in our two series were histologically pure oligodendrogliomas [7,15,16,31] and there was a significant association between frontal lobe location and the allelic loss of 1p and 19q and between temporal lobe location and maintenance of 1p/19q status as previously described in the literature [3,12,33,34]. The differences in the distribution of sex and ages observed between our retrospective and prospective series was not considered a significant source of bias since these two parameters are not correlated to chromosome 1p and 19q status in the literature [15,31]. The analysis of 1p and 19q chromosomal status on retrospective series was performed with Algorithm 1 in concordance with the widely-used ISPO guidelines [28]. Surprisingly this algorithm, although used for FISH analysis of oligodendrogliomas for over 10 years, gives poorly detailed instructions as to the classification of all possible G/R combinations on paraffin embedded tissue section for 1p and 19q into three categories by chromosomal status. A few articles in the literature give more precise definitions for some combinations [15,17,27,29,35] but the definitions are often contradictory, so there is no consensus as to how to classify these G/R combinations. Indeed, according to some studies, combinations 3/3, 4/4 or 5/5 should to be considered as normal status combinations [17,27,35] while for others they indicate polysomy and should be classified as imbalanced status combinations [29]. Likewise, the frequently observed combination 3/2 is considered as an imbalanced combination by some authors [27,28] and as a relative deletion combination for others [16]. Finally, although all tumor cells should be taken into account regardless of their probe signal status, very few instructions exist in the different guidelines on how to classify combinations with no green or no red signals. For this purpose, our retrospective series has the merit of clarifying the importance of certain combinations over others in the determination of 1p/19q status on paraffin sections. Even if both manual and automated analysis reveals a large number of combinations on paraffin sections, due to nuclear truncation artefacts, the majority of these have no importance in the determination of the final chromosomal status, even if the ISPO guidelines for FISH on touch preparations recommend that all combinations should be taken into account [28]. In addition, our retrospective study demonstrates the lack of impact on the final diagnosis for combinations with no G and R signals (0/0) or those without any G signals (G/R: 0/1, 0/2, 0/3 and 0/4). The combination 1/1 is present in 10 to 20% of neoplastic cells, but appeared homogeneously distributed in the three chromosomal status categories and showed no discriminant value. Finally, the combination 3/2 did not appear to be correlated to either deletion status alone [16] or to imbalanced status alone [27–29] in our series and thus showed no discriminant value. At the end of the first phase of our study on the distribution of G/R combinations, we were able to select a reduced number of combinations on which to focus further attention. These 24 selected combinations were highly discriminant for one of the 3 defined chromosomal status, but their respective categories changed sometimes from that reported in the literature. Indeed, the combination 1/2, although not discussed in the literature on touch preparation analysis [28], and usually thought of as a sign of polysomy status, appears strongly linked in our series to normal status category, most likely due to nuclear truncation artifacts of cells with 2/2 combinations rather than a true polysomic status. Although classified in the ISPO guidelines as “non deleted and non imbalanced” status combinations, the combinations 3/3, 4/4 and 5/5 fit better into the imbalanced category, as seen in our study, since they both meet the polysomy criteria [14,16,34,36–38]. Ultimately, our retrospective series allowed us to isolate, from the dozens of combinations observed in neoplastic oligodendroglial cells on paraffin embedded sections, a subset of 24 discriminating combinations, permitting a simplified algorithm (Algorithm 2) which is easy to perform and to compare, and that was validated in our prospective series. The performance of Algorithm 2 on the prospective series was compared to those of Algorithm 1 on the retrospective series. Both algorithms showed a good concordance between manual and automated analysis, regardless of the 3 methods of signal calculation taking into account. These results are in agreement with the previous article on this subject in the literature, which found a complete concordance between manual and automated analysis of oligodendroglial tumors by FISH on touch preparations using the combination method to stratify the results [27].

In our study, both algorithms highlighted the value of the ratio method in the detection of a chromosome arm deletion and its discordant results with combination and combination + ratio methods for the detection of normal or imbalance status. The respective advantages and disadvantages of ratio and combination methods have not been detailed in the literature to our knowledge and there is no clear evidence that it is better to use one method or another. There is no clear consensus in the literature in which method of signal analysis to use. Indeed some authors use the combination method alone [13,16,17,27,28] or the ratio method alone [4,7,10,18,31,39–42] or an association of the two [19,34,36,37,43–48] to determine the chromosomal status of 1p and 19q. Although convergent in many cases, combination and ratio methods are not necessarily superimposable. However, a more detailed analysis of the literature data shows that ratio method is usually used to define the deletion status with a cut off value below 0.8 suggesting that other cases are normal and that it has never been truly defined for imbalanced/polysomic cases. Indeed for the latter, the literature most often identifies cases by the combination method, which appears more adapted to detect small groups of cells with a distinct chromosomal profile, while the ratio method gives an overall average of all examined cells and appears less suited to highlight a particular chromosomal profile in a small group of cells. In our study ratio methods showed a very slight advantage over the combination method in the detection of deleted cases whereas the combination method showed a much greater advantage over the ratio method in the detection of imbalanced cases. This explains why we chose to test and to retain the combination + ratio method, as it appears to give highly satisfactory results in the detection of both deleted and imbalanced cases, especially since recent studies highlight the strong correlation between the presence of an imbalanced tumor cell population and a shorter overall survival [14,38].

Compared to Algorithm 1, Algorithm 2 showed a better concordance for the manual and automated analysis using the method of combinations and combinations + ratio but showed a less satisfactory concordance when using the ratio technique. This difference between the two algorithms lies in the definition of Algorithm 2 in which one of the most frequently encountered combinations (1/2) is classified as normal by the method of combinations although it has a G/R ratio < 1 while combinations 3/3, 4/4 and 5/5 are classified as imbalanced although they have a G/R ratio = 1. Extended to several cells during an analysis, these differences in distribution would explain the differences observed between the two algorithms for ratio method.

Our study showed a good concordance between manual and automated analysis whatever the algorithm or the counting method used but this concordance is not perfect unlike the results described in the only similar study in the literature [27]. This difference between the two studies may be explained by the different tissue analyzed (paraffin embedded tissue versus imprints of frozen tissue), the difference in cut-off values used (30% versus 6%) or the difference between the number of nuclei analyzed (100 versus 200). The extension of our analysis to 200 cells allowed us to obtain a total concordance of 100% and this with a one-way recategorization of the manual findings to match the automated final result using Algorithm 2 but not with Algorithm 1. This can be explained by the fact that the automated analysis is performed on hundred of cells (median varying between 300 to 600 in our series) whereas manual analysis was set in our study at only 100 cells, hence the results of the automated analysis are always closer to reality than the manual analysis. Nevertheless, in majority of our cases (nearly 80%) manual analysis of more than 100 cells would have been unnecessary, and we recommend that it be done only in the rare cases of discordance with the automated counting. Lack of full concordance between automated and manual counting of 200 cells with Algorithm 1 can be explain by the classification of some combinations in an inappropriate chromosomal subgroup which persists regardless of the number of cells analyzed.

Algorithm 2 was also applied to automated analysis of a series of gliomas from an outside institution with a very satisfactory concordance with the initial diagnosis (89% of concordance with a κ = 0.8). All discordant cases could be accounted for by an important fading of the original fluorescent signal.

The implementation of our algorithm in software at our institution was very easy to do, only 27 instructions had to be added in the Metafer software program defining each combination and its subgroup. Automated analysis showed a consequent gain of time compared to manual analysis since in our experience the software performed the analysis on many more cells (up to 10 times more) with less much time (2 to 5 times less). The very good concordance between manual and automated analysis led us to cancel the second manual control that was previously systematically performed in our institution for the concordant cases, which led to a consequent medical and technical time cost saving. Moreover, automated analysis allows to save all fluorescent probe signal images into the computer memory, which avoids slide storage at -20°C or -80°C for archival purpose and all technical and cost problem inherent to this storage. It also avoids fluorescence signal fading such as that observed in a large proportion of the external archival slides analyzed in our study. Numerical stored images allow an easy and fast inter-institutional data exchange and a very easy mutual control on two different platform softwares.

## Conclusion

Automated analysis of 1p and 19q status in oligodendroglial tumors can be achieved in the majority of institutions using the FISH technique on paraffin embedded tissue sections. Despite their widespread diffusion on commercially available FISH platforms, automated software is not mentioned in the oligodendroglial tumor literature and there are no specific guidelines as to how to use it. In this study we propose a simplified G/R combination sequence algorithm with 24 G/R combinations highly correlated to chromosomal 1p and/or 19q status, and a combination + ratio method of analysis, which gives the most satisfactory concordance between manual and automated analysis. Using this algorithm allows a high concordance between automated and manual analysis on 100 tumor cells in a large majority of cases and and additional counting of a further 100 cells leads to a total concordance between the two analysis. Our algorithm was successfully validated on a series from an outside institution which should facilitate multicentric evaluation and standardization of 1p/19q assessment in gliomas and allowed gain in analysis time and cost.

## Supporting Information

S1 Table IRB Waiver form for this study from the Research Ethics Committee of the Centre Hospitalier Universitaire de Québec. (PDF)

S2 Table Validation of Algorithm 2 on a series from an outside institution—Concordance results. (DOCX)
