# Supplementary material for: Automated Analysis of 1p/19q Status by FISH in Oligodendroglial Tumors: Rationale and Proposal of an Algorithm
Source: PLoS One. 2015 Jul 2;10(7):e0132125. doi: 10.1371/journal.pone.0132125 (PMC4489714; doi:10.1371/journal.pone.0132125)
Supplement: S1 Table — (PDF) [file pone.0132125.s001.pdf]

CHU de Québec

Par courrier électronique seulement

Le 13 mai 2015

Docteur Stephan Saikali
Pathologiste
a/s de Michelle Orain
Hôpital Saint-Sacrement du CHU de Québec – Université Laval
1050 chemin Sainte-Foy,
Québec (QC) G1A 4L8

Objet : Assurance qualité / Évaluation de la méthodologie
2016-2455 : Analyse FISH automatisée

Docteur Saikali,

Votre demande datée du 5 mai 2015 concernant la nécessité ou non de faire approuver votre projet de recherche par le Comité d'éthique de la recherche a été évaluée. Après l'avoir analysé, il appert que, selon l'Énoncé de politique des trois Conseils, *Éthique de la recherche avec des êtres humains, 2010 (EPTC2, article 2,5),* votre devis de recherche n'est pas considéré comme un projet de recherche nécessitant l'approbation d'un comité d'éthique de la recherche. Conséquemment le CÉR du CHU de Québec n'a pas à approuver votre projet afin qu'il puisse se dérouler au CHU de Québec.

Toutefois, le devis présenté apparaît conforme aux principes qui régissent l'éthique de la recherche. Les considérations habituelles pour l'accès aux dossiers et le respect de la confidentialité selon les règles des Bonnes Pratiques Cliniques sont applicables.

Les informations recueillies ne devront servir qu'aux seuls objectifs de la présente activité d'évaluation et ne devront pas faire l'objet d'une banque de données ou être utilisées ultérieurement à d'autres fins. De plus, aucun contact avec les patients ou les parents (dans le cas de personnes mineures) ne sera effectué dans le cadre de cette activité d'évaluation. Le CÉR recommande de procéder à l'anonymisation irréversible une fois que les analyses seront complétées afin de permettre une protection maximale de la confidentialité.

Je vous prie d'agréer, Docteur, l'expression de mes sentiments les meilleurs.

Ana Marin, Ph.D.
Présidente, profil A
Comité d'éthique de la recherche du CHU de Québec

AM/vl
