# Supplementary material for: Automated Analysis of 1p/19q Status by FISH in Oligodendroglial Tumors: Rationale and Proposal of an Algorithm
Source: PLoS One. 2015 Jul 2;10(7):e0132125. doi: 10.1371/journal.pone.0132125 (PMC4489714; doi:10.1371/journal.pone.0132125)
Supplement: S2 Table — (DOCX) [file pone.0132125.s002.docx]

| **S2 Table**. Validation of Algorithm 2 on an external series - Concordance Results. | | | | | | | |
| --- | --- | --- | --- | --- | --- | --- | --- |
|  |  |  | **1p status** | |  | **19q status** | |
| **Patient** | **Histology and Grade** |  | **Algorithm results** | **Initial results** |  | **Algorithm results** | **Initial results** |
| 1 | PA (I) |  | **N** | **N** |  | **N** | **N** |
| 2 | A III |  | **N** | **N** |  | **I** | **I** |
| 3 | O II |  | **D** | **D** |  | **D** | **D** |
| 4 | O II |  |  |  |  | **D** | **D** |
| 5 | O II |  | **D** | **D** |  | **D** | **D** |
| 6 | O II |  |  |  |  | **D** | **D** |
| 7 | O II |  | **D** | **D** |  | **D** | **D** |
| 8 | O II |  | **D** | **D** |  | **D** | **D** |
| 9 | O III |  | **N** | **D** |  | **N** | **D** |
| 10 | OA II |  | **I** | **I** |  | **I** | **I** |
| 11 | OA II |  | **D** | **D** |  | **D** | **D** |
| 12 | OA II |  | **N** | **N** |  | **N** | **N** |
| 13 | OA II |  | **I** | **N** |  | **I** | **N** |
| 14 | OA II |  |  |  |  | **D** | **D** |
| 15 | OA III |  | **N** | **N** |  | **N** | **N** |
| 16 | OA III |  |  |  |  | **D** | **D** |
| 17 | OA III |  | **I** | **I** |  | **I** | **I** |
| 18 | OA III |  | **N** | **N** |  | **N** | **N** |
| 19 | OA III |  | **D** | **D** |  | **D** | **D** |
| 20 | OA III |  | **I** | **I** |  | ***** | ***** |
| 21 | OA III |  | **N** | **D** |  | **N** | **D** |
| 22 | OA III |  | **D** | **N** |  | **D** | **D** |
| 23 | OA III |  | **I** | **I** |  | **I** | **I** |
| 24 | OA III |  | **D** | **D** |  | **D** | **D** |
| 25 | OA III |  | **D** | **D** |  | **D** | **D** |
| 26 | OA III |  | **I** | **I** |  | **D** | **D** |
| 27 | OA III |  | **D** | **D** |  | **I** | **D** |
| 28 | OA IV |  | **N** | **N** |  | **N** | **N** |
| 29 | OA IV |  | **N** | **D** |  | **N** | **N** |
| 30 | GBMO |  | **I** | **I** |  | **I** | **I** |
| 31 | GBMO |  | **I** | **I** |  | ***** | ***** |
| 32 | GBMO |  | **N** | **N** |  | **N** | **N** |
| 33 | GBMO |  | **N** | **N** |  | **N** | **N** |
| 34 | GBMO |  | **I** | **I** |  | **I** | **I** |
| 35 | GBMO |  |  |  |  | **N** | **N** |
| 36 | DNET (I) |  | **N** | **N** |  | **N** | **N** |
| Discordant results, Algorithm not applicable, ***** Untested cases.  (D) deletion, (N) normal, (I) imbalance.  (PA) Pilocytic astrocytoma, (O) Oligodendroglioma, (OA) Oligoastrocytoma,  (GBMO) glioblastoma with oligodendroglial component  (DNT) Dysembryoplastic Neuroepithelial Tumour | | | | | | | |
